# The Contribution of Non-Conventional T Cells and NK Cells in the Mycobacterial-Specific IFNγ Response in Bacille Calmette-Guérin (BCG)-Immunized Infants

**DOI:** 10.1371/journal.pone.0077334

**Published:** 2013-10-03

**Authors:** Christel Zufferey, Susie Germano, Binita Dutta, Nicole Ritz, Nigel Curtis

**Affiliations:** 1 Murdoch Children’s Research Institute, Parkville, Australia; 2 Department of Paediatrics, the University of Melbourne, Parkville, Australia; 3 The Royal Children’s Hospital, Melbourne, Parkville, Australia; Federal University of São Paulo, Brazil

## Abstract

**Background:**

The *Mycobacterium bovis* Bacille Calmette-Guérin (BCG) vaccine is given to >120 million infants each year worldwide. Most studies investigating the immune response to BCG have focused on adaptive immunity. However the importance of TCR-gamma/delta (γδ) T cells and NK cells in the mycobacterial-specific immune response is of increasing interest.

**Methods:**

Participants in four age-groups were BCG-immunized. Ten weeks later, in vitro BCG-stimulated blood was analyzed for NK and T cell markers, and intracellular IFNgamma (IFNγ) by flow cytometry. Total functional IFNγ response was calculated using integrated median fluorescence intensity (iMFI).

**Results:**

In infants and children, CD4 and CD4-CD8- (double-negative (DN)) T cells were the main IFNγ-expressing cells representing 43-56% and 27-37% of total CD3+ IFNγ+ T cells respectively. The iMFI was higher in DN T cells compared to CD4 T cells in all age groups, with the greatest differences seen in infants immunized at birth (p=0.002) or 2 months of age (p<0.0001). When NK cells were included in the analysis, they accounted for the majority of total IFNγ-expressing cells and, together with DN Vδ2 γδ T cells, had the highest iMFI in infants immunized at birth or 2 months of age.

**Conclusion:**

In addition to CD4 T cells, NK cells and DN T cells, including Vδ2 γδ T cells, are the key populations producing IFNγ in response to BCG immunization in infants and children. This suggests that innate immunity and unconventional T cells play a greater role in the mycobacterial immune response than previously recognized and should be considered in the design and assessment of novel tuberculosis vaccines.

## Introduction

The *Mycobacterium bovis* Bacille Calmette-Guérin (BCG) vaccine is given to more than 120 million children worldwide each year and remains a key intervention in the prevention of tuberculosis (TB) [1]. In infants it provides approximately 80% protection against severe forms of TB [2].

Understanding the immune response to BCG immunization provides important information in the search for immunological correlates of protection against TB. Surrogate biomarkers of protection against TB remain elusive but are important for the development of improved TB diagnostics and vaccines.

Most studies investigating the immune response to BCG and protection against TB have investigated adaptive immunity [3–5]. In recent years there has been increasing recognition of the importance of the innate immune response in early neonatal life [6–9]. T cells with a gamma-delta (γδ) TCR and NK cells play a key role in innate immunity. These cells increase in frequency during foetal development and represent major cell subsets in cord blood [10–12]. To date, only few studies have investigated the innate immune response to BCG immunization in infants.

We have previously reported the CD4 and CD8 T cell responses 10 weeks after BCG immunization [3,13]. In this study we used samples from the same studies to investigate the role of CD4^-^CD8^-^ double negative (DN) T cells, Vδ2 γδ T cells and NK cells in the mycobacterial-specific IFNgamma (IFNγ) response after BCG immunization.

## Methods

### Ethics Statement

The study was approved by the Human research ethics committees at the Mercy Hospital for Women (R07/16), the Royal Children’s Hospital (26191) and The University of Melbourne (0828435). Written informed consent was obtained from participants or parents.

### Study participants

Infants were recruited at the Mercy Hospital for Women in Melbourne as part of a previous study [3]. Children aged between 10 and 24 months that needed BCG immunization for travel to high TB-prevalence countries were recruited at the Royal Children’s Hospital, Melbourne [13]. Adult volunteers were recruited from University of Melbourne medical students aged between 22 and 27 years who planned to work during their elective overseas in high TB-prevalence countries [13].

### BCG vaccine

BCG Denmark, SSI-1331 (Statens Serum Institute, Copenhagen, Denmark) was used to immunize infants in the first week of life or at 2 months of age [3]. BCG Connaught (Sanofi Pasteur, Toronto, Canada) was used to immunize children older than 2 months and adult participant [13]. BCG vaccine was administered intradermally in the left deltoid region.

### Whole blood assay

Blood was obtained 10 weeks after immunization for *in vitro* assays. To measure cytokine production, whole blood was stimulated with BCG (1.6 x 10^6^ CFU/ml of the same BCG vaccine strain used for immunization reconstituted with Roswell Park Memorial Institute medium) for 7 hours at 37°C in the presence of co-stimulatory antibodies CD49d and CD28 (1 µg/ml each; both from BD Biosciences, San Jose, USA) or left unstimulated (nil control). After addition of brefeldin A (Sigma-Aldrich, St. Louis, USA) at a concentration of 10 µg/ml cells were incubated for 5 additional hours, harvested with 2 mM EDTA (Sigma-Aldrich) then fixed with FACS lysing solution (BD Biosciences) and stored at -80 °C.

### Flow cytometry

Stored blood samples were thawed at 37 °C, permeabilized with Perm 2 buffer for 10 minutes (BD Biosciences) and stained for 30 minutes in the dark with the following anti-human antibodies: CD4-allophycocyanin-efluor 780 (clone SK3; eBioscience, San Diego, USA), CD8-Qdot605 (3B5; Invitrogen, Carlsbad, USA), CD3-Pacific blue (UCHT1), Vδ2 TCR-PE (B6), CD56-allophycocyanin (NCAM 16.2), IFNγ-AlexaFluor 700 (B27) (all BD Biosciences). Cells were acquired using LSRII flow cytometer (BD Biosciences) and analyzed with FlowJo 8.8 (TreeStar, Ashland, USA) and Prism 5 (GraphPad Software, La Jolla, USA). Cytometer setup and tracking beads (BD Biosciences) were used to define LSRII baseline and run daily measurements. CompBeads set anti-mouse Igk (BD Biosciences) was used to optimize fluorescence compensation settings. For each sample, a minimum of 10^6^ cells was acquired. Proportions of BCG-induced cytokine producing cells were analyzed after background correction by subtracting the nil control sample values. Median fluorescence intensity (MFI) was calculated using FlowJo. MFI of BCG-stimulated samples was background corrected by subtracting the MFI of nil control samples. The total functional response of a cell population producing IFNγ is expressed as the integrated MFI (iMFI) and was calculated by multiplying the frequency of IFNγ-expressing T cells by the related MFI as described previously [14].

**Figure 1:**
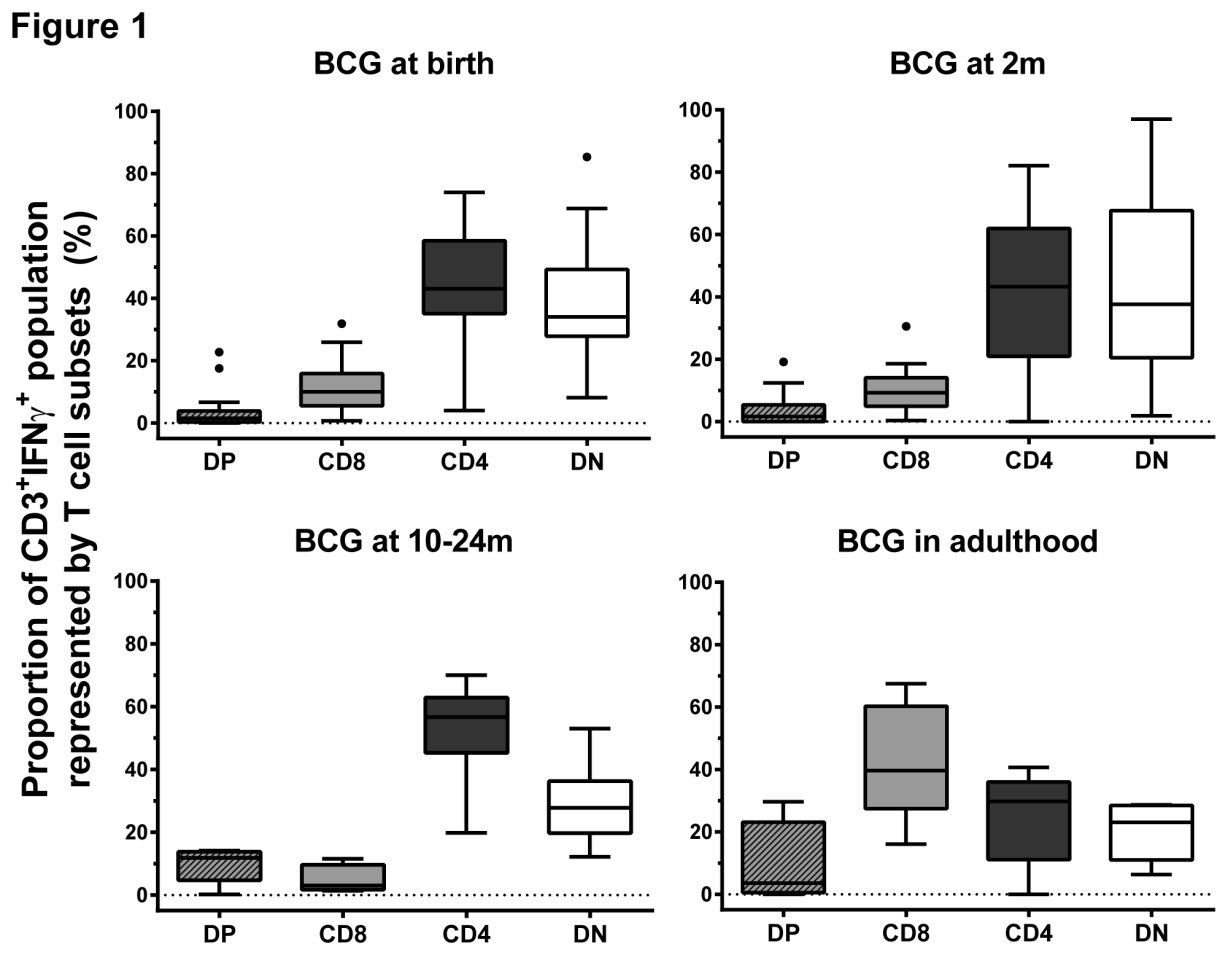
CD4 and DN (CD4^-^CD8^-^) T cells are the main IFNγ-expressing subsets in blood taken from infants 10 weeks after BCG immunization. Box plots (with lower quartile, median and upper quartile, Tukey whiskers) of the proportion of DP, CD8, CD4, DN T cell subsets within the IFNγ^+^ expressing cells in individuals given BCG at birth (n=28), at 2 months of age (2m; n=27), between 10 and 24 months of age (10-24m; n=7) and in adulthood (n=5). DN: CD4^-^CD8^-^ double negative T cells. DP: CD4^+^CD8^+^ double positive T cells.

**Figure 2:**
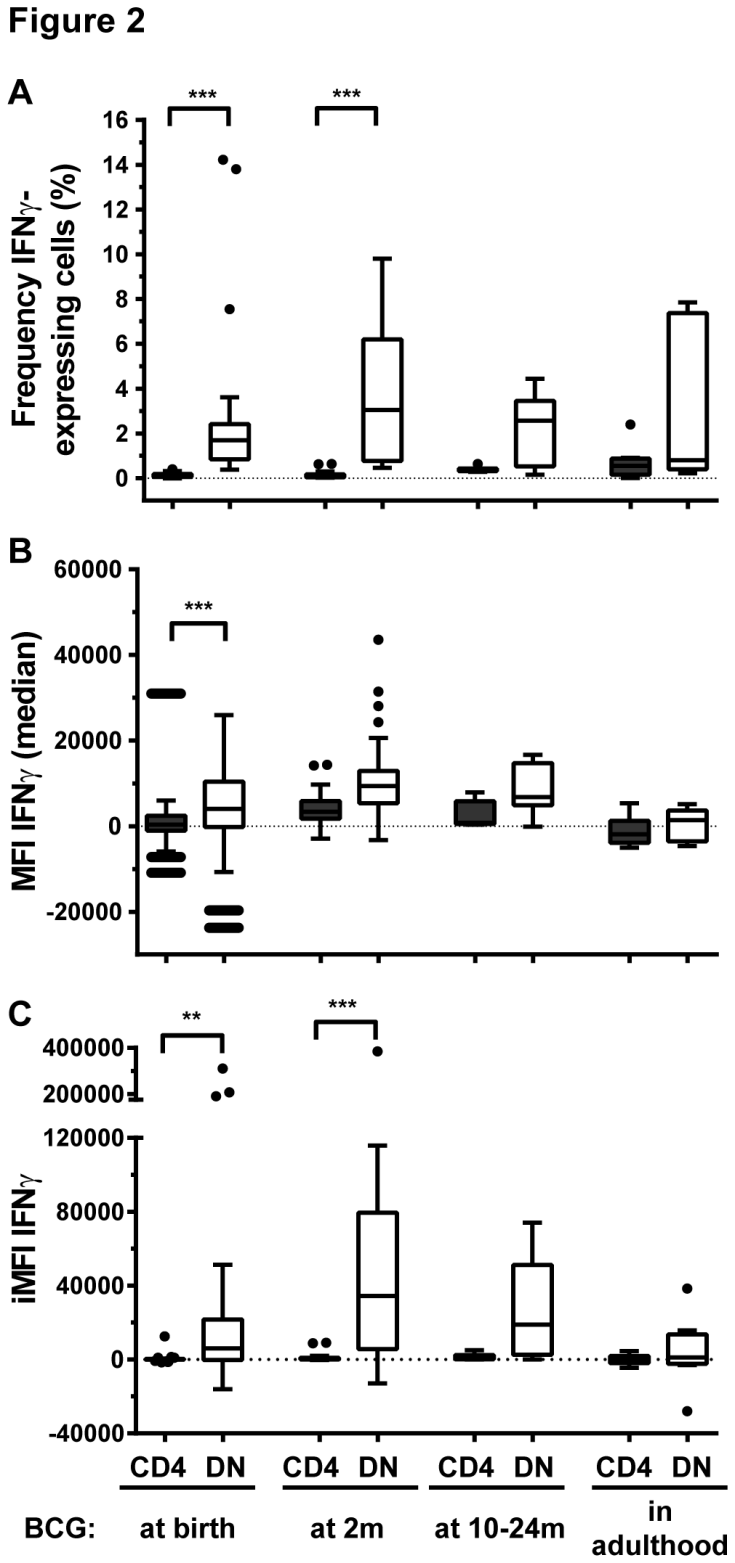
DN (CD4^-^CD8^-^) T cells have a higher IFNγ functional response than CD4 T cells in blood taken from infants 10 weeks after BCG immunization. (A) Frequency and (B) median fluorescence intensity (MFI) of IFNγ-expressing CD4 (grey bars) and DN T cells (white bars), and (C) IFNγ total functional response (iMFI) in individuals given BCG at birth (n=28), at 2 months of age (2m; n=26), between 10 and 24 months of age (10-24m; n=7) and in adulthood (n=9). Box plots with lower quartile, median, upper quartile and Tukey whiskers are shown. **: p<0.001, ***: p<0.0001. DN: CD4^-^CD8^-^ double negative T cells.

**Figure 3:**
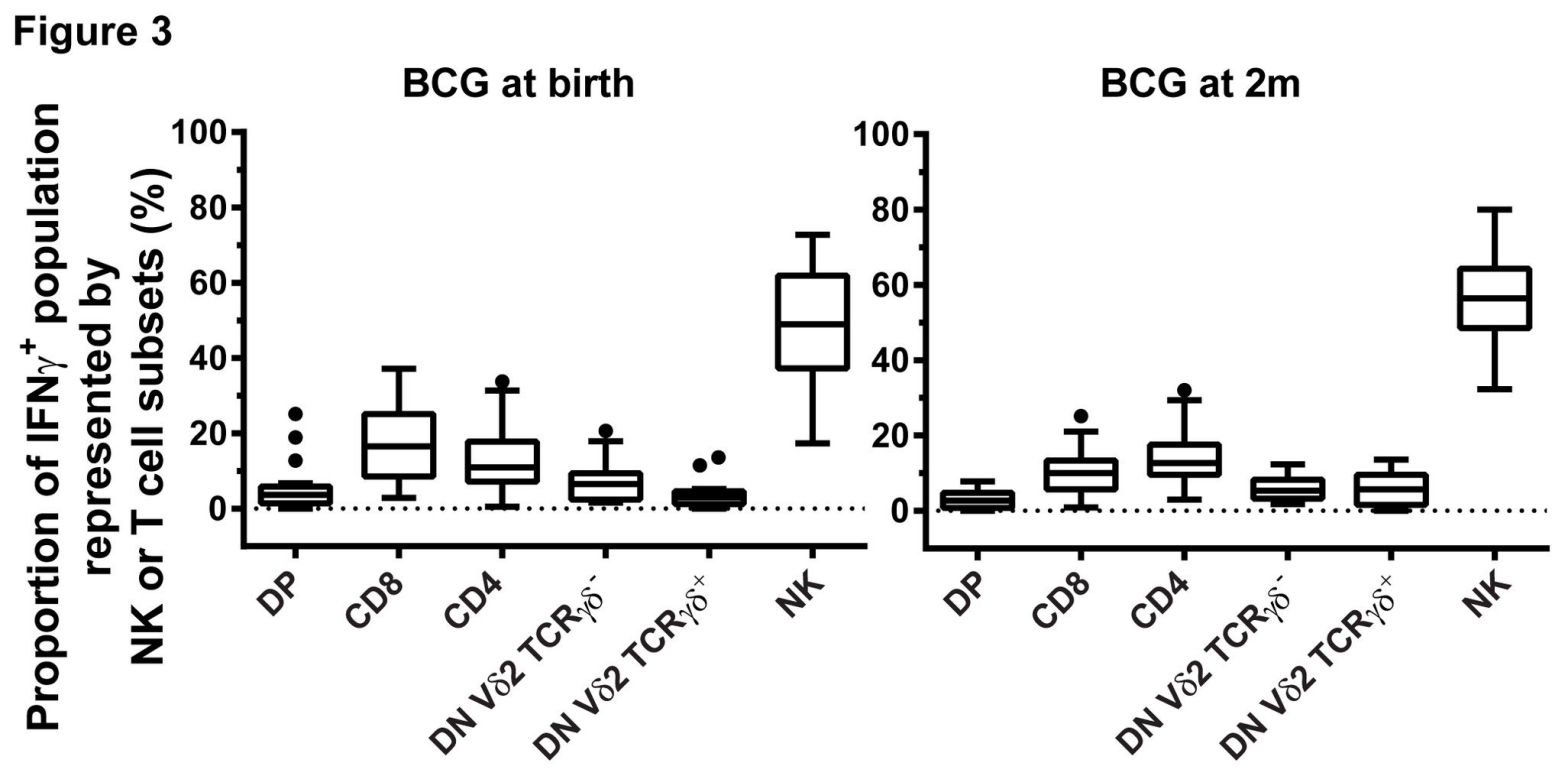
NK, DN Vδ2 TCRγδ^-^ and DN Vδ2 TCRγδ^+^ T cells represent two-thirds of measured IFNγ-expressing cells in blood taken from infants 10 weeks after BCG immunization given at birth (n=20) or at 2 months of age (2m; n=23). Box plots (with lower quartile, median and upper quartile, Tukey whiskers) of the proportion of DP, CD8, CD4, DN Vδ2 TCRγδ^-^, DN Vδ2 TCRγδ^+^ and NK cells within the combined NK IFNγ^+^ and CD3^+^ IFNγ^+^ population. DN: CD4^-^CD8^-^ double negative T cells. DP: CD4^+^CD8^+^ double positive T cells.

**Figure 4:**
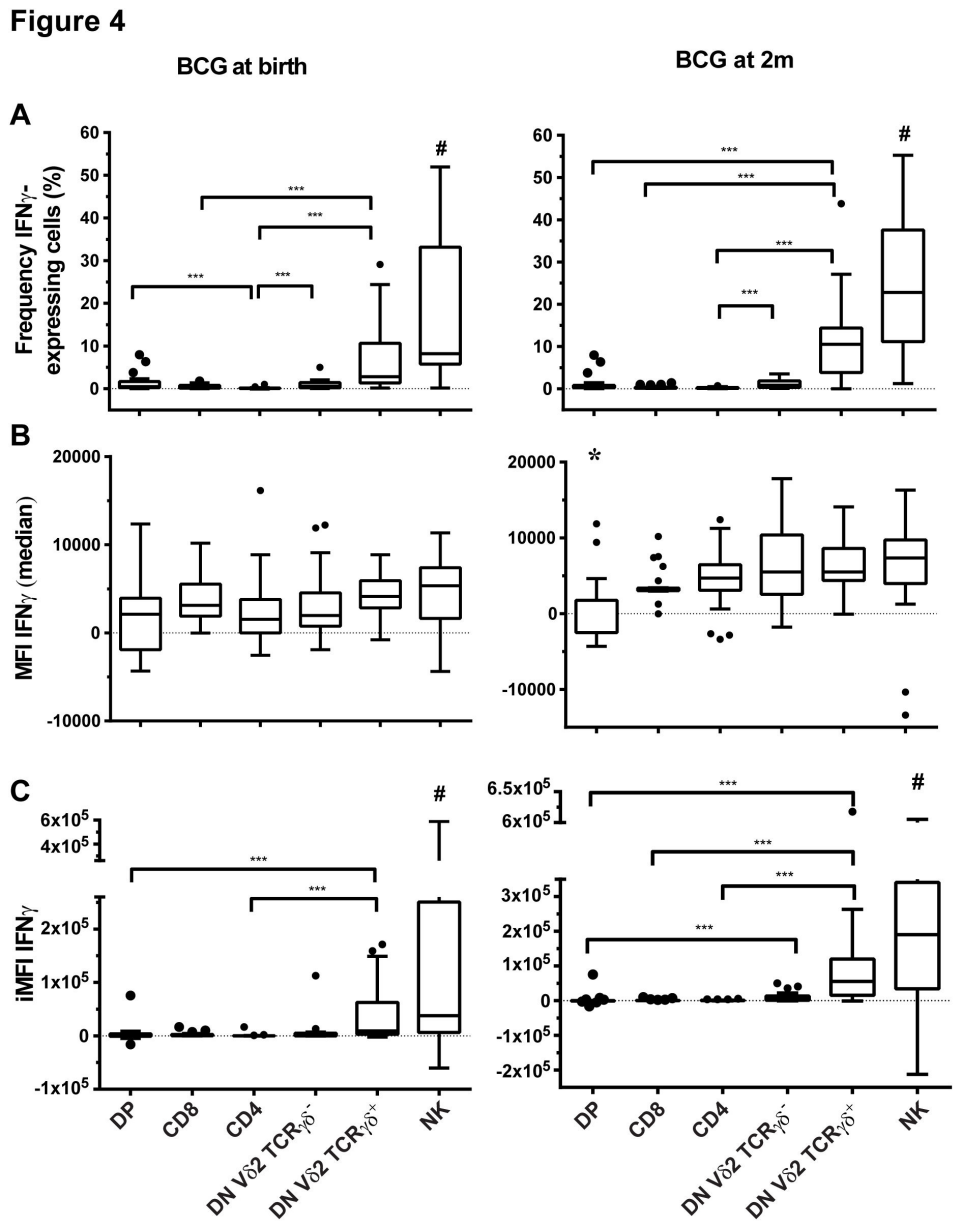
NK and DN Vδ2 TCRγδ^+^ T cells have the highest IFNγ functional response in blood taken from infants 10 weeks after BCG immunization given at birth (n=21) or at 2 months of age (2m; n=25). (A) Frequency and (B) median fluorescence intensity (MFI) of IFNγ-expressing DP, CD8, CD4, DN Vδ2 TCRγδ^-^, DN Vδ2 TCRγδ^+^ T cells and NK cells, and (C) IFNγ total functional response (iMFI) in those subsets. Box plots with lower quartiles, median, upper quartiles and Tukey whiskers are shown. ***: p<0.0001. #: NK cells are different from all subsets except DN Vδ2 TCRγδ^+^ with a p ≤ 0.0012. *: DP MFI is different from all subset MFI except CD8 with a p ≤ 0.0007. DN: CD4^-^CD8^-^ double negative T cells. DP: CD4^+^CD8^+^ double positive T cells.

Results shown in Figures 1 and 2 depict re-analyzed data from samples used in previous studies [3,13]. The following conjugated anti-human antibodies were used in these samples: CD3 PerCP-Cyanin5.5 (SK7), CD4 FITC (RPA-T4), CD8 AlexaFluor-700 (RPA-T8) and IFNγ PE-Cyanin7 (4S.B3) (all BD Biosciences). A hierarchical gating strategy was used to determine proportions of CD4, CD8, DN and double positive (DP) cells within the CD3^+^IFNγ^+^ population (Figure S1). Only samples with more than 100 cells detected in the CD3^+^IFNγ^+^ gate (Figure 1) or in the combined NK and CD3 IFNγ^+^ gate (Figure 3) were included in the analysis. Only samples with more than 10 cells detected in the CD3^+^CD4^-^CD8^-^IFNγ^+^ gate were included in Figures 2 and 4.

### Statistical analysis

A Kruskal-Wallis test and Dunn’s multiple comparison tests were used to compare groups. If the p-value was less than 0.05, a Wilcoxon signed rank test was done to compare two pairs. Graphs were generated and statistics calculated using Prism 5 (GraphPad Software, La Jolla, USA).

## Results

Participants in four age-groups were immunized with BCG. After 10 weeks, blood samples from participants were stimulated with BCG or left unstimulated (nil control), and the mycobacterium-specific immune response was measured by flow cytometry. In children below two years of age, DN T cells represented between 3.4% (n=28) and 7.8% (n=26) of CD3 T cells (Table 1). Despite their small proportion, this subset was responsible for a large share of mycobacterial-specific IFNγ-expressing cells (Figure 1), comparable with the contribution from CD4 T cells. Notably, in contrast to the response observed in children, CD8 T cells were the major contributor of IFNγ-expressing cells in adults (n=5) (Figure 1).

**Table 1:** DN T cells represent approximately 3-9% of total CD3^+^ T cells.

Group given BCG at:	Median (interquartile range) proportion of T cell subset (%)			
	CD4	DN	CD8	DP
Birth (n=28)	73.2 (66.8-78.1)	3.4 (2.2-4.7)	23.7 (16.9-28.6)	0.23 (0.2-0.3)
2 months of age (n=26)	70.8 (59.1-74.7)	4.6 (3.2-5.3)	24.6 (20.5-34.2)	0.15 (0.1-0.2)
10-24 months of age (n=7)	63.6 (57.1-71.3)	7.8 (6-13.1)	22.5 (20-25.5)	0.81 (0.5-0.9)
Adulthood (n=9)	50.8 (46.6-58.5)	9.4 (7.9-12.8)	37.9 (28.7-43.8)	0.44 (0.4-0.8)

DN T cells more frequently expressed IFNγ than CD4 T cells following BCG immunization. At birth (n=28), 1.69% (interquartile range (IQR) 0.8-2.4%) of DN T cells expressed IFNγ compared to 0.08% (IQR 0.04-0.18%) of CD4 T cells, p<0.0001. Similarly at two months of age (n=26), 3% (IQR 0.7-6.2%) of DN T cells expressed IFNγ compared to 0.1% (IQR 0.04-0.16%) of CD4 T cells, p<0.0001 (Figure 2A). Importantly, DN T cells also showed a higher IFNγ-producing capacity (median fluorescence intensity (MFI)) than CD4 T cells and the total functional IFNγ response (combining frequency of IFNγ-expressing cells and MFI) was higher in DN T cells than in CD4 T cells (Figure 2B and 2C).

In a next step, we analyzed the IFNγ expression of NK cells and the phenotypic subgroups of DN T cells (for this, only samples from infants BCG-immunized at birth and two months of age were available [3]). As shown in the gating strategy (Figure S2), NK cells were chosen from the CD56^+^CD3^-^ population and DN T cells were selected from the CD56^-^CD3^+^CD4^-^CD8^-^ population and then analyzed for their Vδ2 TCRγδ expression. The proportions of Vδ2 γδ T cells within the DN T cell population were 12.6% (IQR 6.5-19.7%) and 7.9% (IQR 4.8-12.3%) in blood taken from infants immunized with BCG at birth (n=21) and at two month of age (n=25) respectively. NK cells, DN Vδ2 TCRγδ^+^ and DN Vδ2 TCRγδ^-^ T cells represented a substantial proportion of IFNγ-expressing cells, with NK cells alone contributing to more than half the measured total IFNγ-expressing cells in both age groups (Figure 3).

Up to 23% of NK cells and 11% of DN Vδ2 TCRγδ^+^ T cells expressed IFNγ compared to less than 1% of double positive (DP), CD8, CD4 and DN Vδ2 TCRγδ^-^ T cells expressing IFNγ in infants BCG-immunized at birth (n=21) and at 2 months of age (n=25) (Figure 4A). The IFNγ-expressing capacity was comparable in all subsets with the exception of DP T cells, which had a lower IFNγ MFI in infants immunized at two months of age (Figure 4B). Consequently, the greatest IFNγ functional response was measured in NK cells and DN Vδ2 TCRγδ^+^ T cells (Figure 4C). Notably, CD4 T cells were not major contributors to the total IFNγ functional response (Figure 4C).

## Discussion

Our study is the first to investigate in detail the importance of NK cells, γδ T cells and DN T cells in the mycobacterial-specific IFNγ response following BCG immunization in infants. We found that the key populations producing IFNγ in response to BCG in infants and children were NK cells and DN T cells, including Vδ2 γδ T cells, rather than CD4 T cells. This highlights the potential importance of the innate immune response and unconventional T cells in the immunoprotective response to BCG.

Previous studies of the immune response to BCG have largely focused on cell-mediated immunity. A CD4 T cell (Th1-type) response associated with IFNγ expression and cytotoxic activity is observed in infants and children after BCG immunization [3,13,15–19]. BCG also induces dendritic cell maturation and production of IL-12 that leads to Th1 differentiation [20–22]. Activation of CD8 T cells producing IFNγ, TNFα and perforin has also been demonstrated [3,23]. In our study, we found that in BCG-immunized adults, in contrast to infants, CD8 T cells were the main IFNγ-producing cells. This suggests that this subset is a crucial player in the immune response to TB in adults as previously proposed [23]. Another recent study in adults shows that CD4 T cells expressed lower IFNγ level than CD8 and DN T cells in TB patients [24] consistent with our results. Although it has been suggested that non-conventional T cells and innate immunity play a role in the response to BCG immunization [25], this aspect of TB immunity has been less well investigated.

Our results show that while DN T cells represent only a small proportion of T cells, this subset makes a considerable contribution to the IFNγ response in infants immunized with BCG that is greater than that made by CD4 T cells. These findings are consistent with a previous study in humans showing that DN T cells represent approximately 4% of T cells in PBMC and express 3 to 4 times more IFNγ than CD4 T cells [26]. It has been suggested that DN T cells play an immunoregulatory role as they can express perforin and suppress cytotoxic CD8 T cells [26]. In humans, DN T cells suppress CD4 and CD8 T cell responses [27]. Similarly in mice, DN T cells kill CD4 T cells, B cells and NK cells and down-regulate co-stimulatory molecules on mature dendritic cells thus contributing to immune tolerance [28]. In simian immunodeficiency virus infection, DN T cells develop CD4 T cell functions that parallel the loss of CD4 T cells and protect against viral dissemination [29]. DN T cells are also involved in the mycobacterial-specific immune response in mice [30,31] and develop a memory phenotype, potentially contributing to effective protection [30].

Within the DN T cell population, γδ T cells have long been known to constitute a “first line of defense” linking innate and adaptive immunity [32,33]. Their presence is necessary for the expansion of CD4 T cells and they can also act as antigen-presenting cells and cross-present antigen to CD8 T cells [34,35]. In the early 1990s, γδ T cells were shown to be activated by phosphoantigens, which are abundant in *Mycobacterium tuberculosis* (MTB) [36,37]. In animal studies in mice and pigs immunized with attenuated MTB or BCG, γδ T cells are activated, expanded and express IFNγ[38–40] These cells have cytotoxic activity for BCG-infected macrophages and are necessary to prime antigen-specific CD8 T cell responses through the enhanced production of IL-12 by lung dendritic cells [39,40]. TCRγδ T cell-deficient mice infected with BCG had markedly reduced IFNγ production, suggesting a role in immunity to BCG [39,41,42]. In neonates, when a mature TCRαβ immune system is still lacking, it has been proposed that γδ T cells are crucial for protection against infections [43]. Human γδ T cells have been shown to produce IFNγ when BCG-stimulated *in vitro* [41] and γδ T cells from BCG-immunized infants expand to comprise 60% of total T cells after *in vitro* restimulation [44]. However, the relationship between γδ T cells and protection is uncertain. In infants immunized with BCG at birth, the frequency of IFNγ-producing γδ T cells after immunization did not correlate with protection against TB [45]. In contrast, in patients with severe TB, the frequency of total DN T cells was increased compared to healthy donors, but the DN γδ T cells frequency was reduced. However, both DN and DN γδ T cells expressed IFNγ in patients with moderate disease suggesting a role in the immune response to TB [24]. TB patients with mild disease have a greater γδ T cell frequency compared to patients with advanced pulmonary and miliary TB, and therefore these cells may correlate with protective immunity [46].

NK cells are major players in the innate immune response and their function during MTB infection has increasingly been investigated in the last decade. In BCG-immunized mice, NK cells play a key role in the control of bacterial replication and enhance T cell responses mediated by the secretion of IL-22 and IFNγ [47]. In addition, IFNγ produced by NK cells is crucial for the regulation of T cell-independent resistance to MTB and neutrophil recruitment in lungs of MTB-infected mice [48]. In humans, NK cells produce IFNγ, perforin and granzyme A when stimulated with BCG or PPD [49–51]. It has recently been shown that BCG induces the maturation of NK cells isolated from umbilical cord blood and enhances their cytotoxic activity against immature dendritic cells, suggesting a role in shaping adaptive immunity [52]. NK cells also play a major role in protection against TB by lysis of MTB-infected monocytes and enhancement of CD8 T cell effector functions [53]. Furthermore, in patients with active TB, NK cell activity was diminished [53].

One potential limitation of our study is that different BCG vaccine strains were used for immunization. BCG-Connaught was the licensed vaccine strain for routine immunization in Australia during the study period, while BCG-Denmark was used in the randomized study. No study has compared the *in vitro* immune response to these two vaccines in humans, but a study in mice showed comparable proportions of cytokine-producing CD4 and CD8 T cells in the lungs after immunization with either BCG-Connaught or Denmark [54].

The development of new improved TB vaccines is one of the *WHO Stop TB* priorities, and vaccines that rely on boosting BCG at birth are the most advanced. In a recent randomized controlled trial, the novel boosting vaccine MVA85A failed to show protection of infants despite having shown good mycobacterial-specific adaptive immune responses in previous trials [54]. This underlines the importance of investigating the effects of BCG on early life anti-mycobacterial immunity and the potential importance of other cells such as unconventional T cells and NK cells.

Our results highlight an important role for both DN Vδ2 γδ T cells and NK cells in the mycobacterial-specific IFNγ response to BCG immunization in infants. Recent studies in both mice [55] and humans [24,45] suggest there is not a simple relationship between IFNγ production from T cells and protection against TB. However, our study supports the concept that the role of the innate immune response and unconventional T cells should be considered in future investigation of the immunoprotective function of BCG and potential new TB vaccines.

## Supporting Information

Figure S1 **Gating strategy to select IFNγ-expressing cells within the CD3 T cell population.** The IFNγ positive gate was set using Nil-stimulated samples (top right panel). In BCG-stimulated samples (bottom panels), CD8 and CD4 expression was then analyzed on CD3^+^ IFNγ^+^ cells.
(TIF)

Figure S2 **Gating strategy to select CD56^+^ NK cells and CD56^-^CD3^+^ T cells.** Within the CD56^-^CD3^+^ cells, DN T cells were further gated into DN Vδ2 TCRγδ^+^ and DN Vδ2 TCRγδ^-^ populations. Bottom panels show IFNγ expression in NK, DN Vδ2 TCRγδ^+^ and DN Vδ2 TCRγδ^-^ cells. Note, for clarity, gating of CD4^+^, CD8^+^ and CD4^+^CD8^+^ T cells within CD56^-^CD3^+^ gate is not shown.
(TIF)

## References

[1] Ritz N, Curtis N (2009) Mapping the global use of different BCG vaccine strains. Tuberculosis 89: 248-251. doi:10.1016/j.tube.2009.03.002. PubMed: 19540166. 19540166

[2] Trunz BB, Fine P, Dye C (2006) Effect of BCG vaccination on childhood tuberculous meningitis and miliary tuberculosis worldwide: a meta-analysis and assessment of cost-effectiveness. Lancet 367: 1173-1180. doi:10.1016/S0140-6736(06)68507-3. PubMed: 16616560. 16616560

[3] Ritz N, Dutta B, Donath S, Casalaz D, Connell TG et al. (2012) The influence of bacille Calmette-Guerin vaccine strain on the immune response against tuberculosis: a randomized trial. Am J Respir Crit Care Med 185: 213-222. doi:10.1164/rccm.201104-0714OC. PubMed: 22071384. 22071384

[4] Davids V, Hanekom WA, Mansoor N, Gamieldien H, Gelderbloem SJ et al. (2006) The effect of bacille Calmette-Guerin vaccine strain and route of administration on induced immune responses in vaccinated infants. J Infect Dis 193: 531-536. doi:10.1086/499825. PubMed: 16425132. 16425132

[5] Hanekom WA, Hughes J, Mavinkurve M, Mendillo M, Watkins M et al. (2004) Novel application of a whole blood intracellular cytokine detection assay to quantitate specific T-cell frequency in field studies. J Immunol Methods 291: 185-195. doi:10.1016/j.jim.2004.06.010. PubMed: 15345316. 15345316

[6] Levy O (2007) Innate immunity of the newborn: basic mechanisms and clinical correlates. Nat Rev Immunol 7: 379-390. doi:10.1038/nri2075. PubMed: 17457344. 17457344

[7] Guilmot A, Hermann E, Braud VM, Carlier Y, Truyens C (2011) Natural killer cell responses to infections in early life. J Innate Immun 3: 280-288. doi:10.1159/000323934. PubMed: 21411972. 21411972

[8] Reikie BA, Adams RC, Ruck CE, Ho K, Leligdowicz A et al. (2012) Ontogeny of Toll-Like Receptor Mediated Cytokine Responses of South African Infants throughout the First Year of Life. PLOS ONE 7: e44763. doi:10.1371/journal.pone.0044763. PubMed: 23028609. 23028609 PMC3441420

[9] Watkins ML, Semple PL, Abel B, Hanekom WA, Kaplan G et al. (2008) Exposure of cord blood to Mycobacterium bovis BCG induces an innate response but not a T-cell cytokine response. Clin Vaccine Immunol CVI 15: 1666-1673. doi:10.1128/CVI.00202-08. 18815231 PMC2583525

[10] Peakman M, Buggins AG, Nicolaides KH, Layton DM, Vergani D (1992) Analysis of lymphocyte phenotypes in cord blood from early gestation fetuses. Clin Exp Immunol 90: 345-350. PubMed: 1385028. 1385028 10.1111/j.1365-2249.1992.tb07953.x PMC1554603

[11] Phillips JH, Hori T, Nagler A, Bhat N, Spits H et al. (1992) Ontogeny of human natural killer (NK) cells: fetal NK cells mediate cytolytic function and express cytoplasmic CD3 epsilon,delta proteins. J Exp Med 175: 1055-1066. doi:10.1084/jem.175.4.1055. PubMed: 1372642. 1372642 PMC2119193

[12] Pérez A, Gurbindo MD, Resino S, Aguarón A, Muñoz-Fernández MA (2007) NK cell increase in neonates from the preterm to the full-term period of gestation. Neonatology 92: 158-163. doi:10.1159/000101567. PubMed: 17429221. 17429221

[13] Ritz N, Strach M, Yau C, Dutta B, Tebruegge M et al. (2012) A comparative analysis of polyfunctional T cells and secreted cytokines induced by Bacille Calmette-Guerin immunisation in children and adults. PLOS ONE 7: e37535. doi:10.1371/journal.pone.0037535. PubMed: 22829867. 22829867 PMC3400612

[14] Darrah PA, Patel DT, De Luca PM, Lindsay RW, Davey DF et al. (2007) Multifunctional TH1 cells define a correlate of vaccine-mediated protection against Leishmania major. Nat Med 13: 843-850. doi:10.1038/nm1592. PubMed: 17558415. 17558415

[15] Ravn P, Boesen H, Pedersen BK, Andersen P (1997) Human T cell responses induced by vaccination with Mycobacterium bovis bacillus Calmette-Guerin. J Immunol 158: 1949-1955. PubMed: 9029137. 9029137

[16] Surekha Rani H, Vijaya Lakshmi V, Sumanlatha G, Murthy KJ (2005) Cell-mediated immune responses in children towards secreted proteins of Mycobacterium bovis BCG. Tuberculosis (Edinb) 85: 89-93. doi:10.1016/j.tube.2004.09.010. PubMed: 15687032. 15687032

[17] Hussey GD, Watkins ML, Goddard EA, Gottschalk S, Hughes EJ et al. (2002) Neonatal mycobacterial specific cytotoxic T-lymphocyte and cytokine profiles in response to distinct BCG vaccination strategies. Immunology 105: 314-324. doi:10.1046/j.1365-2567.2002.01366.x. PubMed: 11918693. 11918693 PMC1782661

[18] Hoft DF, Kemp EB, Marinaro M, Cruz O, Kiyono H et al. (1999) A double-blind, placebo-controlled study of Mycobacterium-specific human immune responses induced by intradermal bacille Calmette-Guerin vaccination. J Lab Clin Med 134: 244-252. doi:10.1016/S0022-2143(99)90204-4. PubMed: 10482309. 10482309

[19] Marchant A, Goetghebuer T, Ota MO, Wolfe I, Ceesay SJ et al. (1999) Newborns develop a Th1-type immune response to Mycobacterium bovis bacillus Calmette-Guerin vaccination. J Immunol 163: 2249-2255. PubMed: 10438968. 10438968

[20] Yokoi T, Amakawa R, Tanijiri T, Sugimoto H, Torii Y et al. (2008) Mycobacterium bovis Bacillus Calmette-Guerin suppresses inflammatory Th2 responses by inducing functional alteration of TSLP-activated dendritic cells. Int Immunol 20: 1321-1329. doi:10.1093/intimm/dxn094. PubMed: 18703465. 18703465

[21] Kim KD, Lee HG, Kim JK, Park SN, Choe IS et al. (1999) Enhanced antigen-presenting activity and tumour necrosis factor-alpha-independent activation of dendritic cells following treatment with Mycobacterium bovis bacillus Calmette-Guerin. Immunology 97: 626-633. doi:10.1046/j.1365-2567.1999.00818.x. PubMed: 10457216. 10457216 PMC2326884

[22] Demangel C, Bean AG, Martin E, Feng CG, Kamath AT et al. (1999) Protection against aerosol Mycobacterium tuberculosis infection using Mycobacterium bovis Bacillus Calmette Guerin-infected dendritic cells. Eur J Immunol 29: 1972-1979. doi:10.1002/(SICI)1521-4141(199906)29:06. PubMed: 10382760. 10382760

[23] Smith SM, Malin AS, Pauline T, Lukey, Atkinson SE et al. (1999) Characterization of human Mycobacterium bovis bacille Calmette-Guerin-reactive CD8+ T cells. Infect Immun 67: 5223-5230. PubMed: 10496899. 10496899 10.1128/iai.67.10.5223-5230.1999 PMC96874

[24] Pinheiro MB, Antonelli LR, Sathler-Avelar R, Vitelli-Avelar DM, Spindola-de-Miranda S et al. (2012) CD4-CD8-alphabeta and gammadelta T Cells Display Inflammatory and Regulatory Potentials during Human Tuberculosis. PLOS ONE 7: e50923. doi:10.1371/journal.pone.0050923. PubMed: 23239994. 23239994 PMC3519797

[25] Abebe F (2012) Is interferon-gamma the right marker for bacille Calmette-Guerin-induced immune protection? The missing link in our understanding of tuberculosis immunology. Clin Exp Immunol 169: 213-219. doi:10.1111/j.1365-2249.2012.04614.x. PubMed: 22861360. 22861360 PMC3444997

[26] Fischer K, Voelkl S, Heymann J, Przybylski GK, Mondal K et al. (2005) Isolation and characterization of human antigen-specific TCR alpha beta+ CD4(-)CD8- double-negative regulatory T cells. Blood 105: 2828-2835. doi:10.1182/blood-2004-07-2583. PubMed: 15572590. 15572590

[27] Voelkl S, Gary R, Mackensen A. (2011) Characterization of the immunoregulatory function of human TCR-alphabeta+ CD4- CD8- double-negative T cells. Eur J Immunol 41: 739-748. doi:10.1002/eji.201040982. PubMed: 21287552. 21287552

[28] Hillhouse EE, Lesage S (2012) A comprehensive review of the phenotype and function of antigen-specific immunoregulatory double negative T cells. J Autoimmun. 10.1016/j.jaut.2012.07.010 22910322

[29] Sundaravaradan V, Mir KD, Sodora DL. (2012) Double-negative T cells during HIV/SIV infections: potential pinch hitters in the T-cell lineup. Curr Opin HIV Aids 7: 164-171. doi:10.1097/COH.0b013e3283504a66. PubMed: 22241163. 22241163 PMC3639317

[30] Cowley SC, Hamilton E, Frelinger JA, Su J, Forman J et al. (2005) CD4-CD8- T cells control intracellular bacterial infections both in vitro and in vivo. J Exp Med 202: 309-319. doi:10.1084/jem.20050569. PubMed: 16027239. 16027239 PMC2212999

[31] Derrick SC, Evering TH, Sambandamurthy VK, Jalapathy KV, Hsu T et al. (2007) Characterization of the protective T-cell response generated in CD4-deficient mice by a live attenuated Mycobacterium tuberculosis vaccine. Immunology 120: 192-206. doi:10.1111/j.1365-2567.2006.02491.x. PubMed: 17076705. 17076705 PMC2265854

[32] Meraviglia S, El Daker S, Dieli F, Martini F, Martino (2011) A gammadelta T cells cross-link innate and adaptive immunity in Mycobacterium tuberculosis infection. Clin Dev Immunol, 2011: 587315 PubMed: 21253470 10.1155/2011/587315 PMC3022180 21253470

[33] Inoue T, Yoshikai Y, Matsuzaki G, Nomoto K (1991) Early appearing gamma/delta-bearing T cells during infection with Calmette Guerin bacillus. J Immunol 146: 2754-2762. PubMed: 1707921. 1707921

[34] Hoft DF, Brown RM, Roodman ST (1998) Bacille Calmette-Guerin vaccination enhances human gamma delta T cell responsiveness to mycobacteria suggestive of a memory-like phenotype. J Immunol 161: 1045-1054. PubMed: 9670986. 9670986

[35] Brandes M, Willimann K, Bioley G, Lévy N, Eberl M et al. (2009) Cross-presenting human gammadelta T cells induce robust CD8+ alphabeta T cell responses. Proc Natl Acad Sci U S A 106: 2307-2312. doi:10.1073/pnas.0810059106. PubMed: 19171897. 19171897 PMC2650152

[36] Tanaka Y, Sano S, Nieves E, De Libero G, Rosa D et al. (1994) Nonpeptide ligands for human gamma delta T cells. Proc Natl Acad Sci U S A 91: 8175-8179. doi:10.1073/pnas.91.17.8175. PubMed: 8058775. 8058775 PMC44568

[37] Pfeffer K, Schoel B, Gulle H, Kaufmann SH, Wagner H (1990) Primary responses of human T cells to mycobacteria: a frequent set of gamma/delta T cells are stimulated by protease-resistant ligands. Eur J Immunol 20: 1175-1179. doi:10.1002/eji.1830200534. PubMed: 2141570. 2141570

[38] Janis EM, Kaufmann SH, Schwartz RH, Pardoll DM (1989) Activation of gamma delta T cells in the primary immune response to Mycobacterium tuberculosis. Science 244: 713-716. doi:10.1126/science.2524098. PubMed: 2524098. 2524098

[39] Dieli F, Ivanyi J, Marsh P, Williams A, Naylor I et al. (2003) Characterization of lung gamma delta T cells following intranasal infection with Mycobacterium bovis bacillus Calmette-Guerin. J Immunol 170: 463-469. PubMed: 12496432. 12496432 10.4049/jimmunol.170.1.463

[40] Caccamo N, Sireci G, Meraviglia S, Dieli F, Ivanyi J et al. (2006) gammadelta T cells condition dendritic cells in vivo for priming pulmonary CD8 T cell responses against Mycobacterium tuberculosis. Eur J Immunol 36: 2681-2690. doi:10.1002/eji.200636220. PubMed: 16981183. 16981183

[41] Naoe M, Ogawa Y, Takeshita K, Morita J, Iwamoto S et al. (2007) Bacillus Calmette-Guerin-pulsed dendritic cells stimulate natural killer T cells and gammadeltaT cells. Int J Urol 14: 532-538; discussion: 10.1111/j.1442-2042.2006.01697.x. PubMed: 17593099. 17593099

[42] Ladel CH, Hess J, Daugelat S, Mombaerts P, Tonegawa S et al. (1995) Contribution of alpha/beta and gamma/delta T lymphocytes to immunity against Mycobacterium bovis bacillus Calmette Guerin: studies with T cell receptor-deficient mutant mice. Eur J Immunol 25: 838-846. doi:10.1002/eji.1830250331. PubMed: 7705416. 7705416

[43] Gibbons DL, Haque SF, Silberzahn T, Hamilton K, Langford C et al. (2009) Neonates harbour highly active gammadelta T cells with selective impairments in preterm infants. Eur J Immunol 39: 1794-1806. doi:10.1002/eji.200939222. PubMed: 19544311. 19544311

[44] Mazzola TN, Da Silva MT, Moreno YM, Lima SC, Carniel EF et al. (2007) Robust gammadelta+ T cell expansion in infants immunized at birth with BCG vaccine. Vaccine 25: 6313-6320. doi:10.1016/j.vaccine.2007.06.039. PubMed: 17643559. 17643559

[45] Kagina BM, Abel B, Scriba TJ, Hughes EJ, Keyser A et al. (2010) Specific T cell frequency and cytokine expression profile do not correlate with protection against tuberculosis after bacillus Calmette-Guerin vaccination of newborns. Am J Respir Crit Care Med 182: 1073-1079. doi:10.1164/rccm.201003-0334OC. PubMed: 20558627. 20558627 PMC2970848

[46] Barnes PF, Grisso CL, Abrams JS, Band H, Rea TH et al. (1992) Gamma delta T lymphocytes in human tuberculosis. J Infect Dis 165: 506-512. doi:10.1093/infdis/165.3.506. PubMed: 1538155. 1538155

[47] Dhiman R, Periasamy S, Barnes PF, Jaiswal AG, Paidipally P et al. (2012) NK1.1+ cells and IL-22 regulate vaccine-induced protective immunity against challenge with Mycobacterium tuberculosis. J Immunol 189: 897-905. doi:10.4049/jimmunol.1102833. PubMed: 22711885. 22711885 PMC3392427

[48] Feng CG, Kaviratne M, Rothfuchs AG, Cheever A, Hieny S et al. (2006) NK cell-derived IFN-gamma differentially regulates innate resistance and neutrophil response in T cell-deficient hosts infected with Mycobacterium tuberculosis. J Immunol 177: 7086-7093. PubMed: 17082625. 17082625 10.4049/jimmunol.177.10.7086

[49] Batoni G, Esin S, Favilli F, Pardini M, Bottai D et al. (2005) Human CD56bright and CD56dim natural killer cell subsets respond differentially to direct stimulation with Mycobacterium bovis bacillus Calmette-Guerin. Scand J Immunol 62: 498-506. doi:10.1111/j.1365-3083.2005.01692.x. PubMed: 16316416. 16316416

[50] Semple PL, Watkins M, Davids V, Krensky AM, Hanekom WA et al. (2011) Induction of granulysin and perforin cytolytic mediator expression in 10-week-old infants vaccinated with BCG at birth. Clin Dev Immunol, 2011: 2011: 438463. PubMed: 21234358 10.1155/2011/438463 PMC3018618 21234358

[51] Smith SG, Lalor MK, Gorak-Stolinska P, Blitz R, Beveridge NE et al. (2010) Mycobacterium tuberculosis PPD-induced immune biomarkers measurable in vitro following BCG vaccination of UK adolescents by multiplex bead array and intracellular cytokine staining. BMC Immunol 11: 35. doi:10.1186/1471-2172-11-35. PubMed: 20609237. 20609237 PMC2910033

[52] Marras F, Bozzano F, Bentivoglio G, Ugolotti E, Biassoni R et al. (2012) Receptor modulation and functional activation of human CD34+ Lin- -derived immature NK cells in vitro by Mycobacterium bovis Bacillus Calmette-Guerin (BCG). Eur J Immunol 42: 2459-2470. doi:10.1002/eji.201242375. PubMed: 22736333. 22736333

[53] Vankayalapati RaB PF (2009) Innate and adaptive immune responses to human Mycobacterium tuberculosis infection. Tuberculosis (Edinb) 89: S77-S80. doi:10.1016/S1472-9792(09)70018-6. 20006312

[54] Castillo-Rodal AI, Castañón-Arreola M, Hernández-Pando R, Calva JJ, Sada-Díaz E et al. (2006) Mycobacterium bovis BCG substrains confer different levels of protection against Mycobacterium tuberculosis infection in a BALB/c model of progressive pulmonary tuberculosis. Infect Immun 74: 1718-1724. doi:10.1128/IAI.74.3.1718-1724.2006. PubMed: 16495544. 16495544 PMC1418655

[55] Connor LM, Harvie MC, Rich FJ, Quinn KM, Brinkmann V et al. (2010) A key role for lung-resident memory lymphocytes in protective immune responses after BCG vaccination. Eur J Immunol 40: 2482-2492. doi:10.1002/eji.200940279. PubMed: 20602436. 20602436

