# Supplementary figures and images for: The Contribution of Non-Conventional T Cells and NK Cells in the Mycobacterial-Specific IFNγ Response in Bacille Calmette-Guérin (BCG)-Immunized Infants
Source: PLoS One. 2013 Oct 3;8(10):e77334. doi: 10.1371/journal.pone.0077334 (PMC3789697; doi:10.1371/journal.pone.0077334)

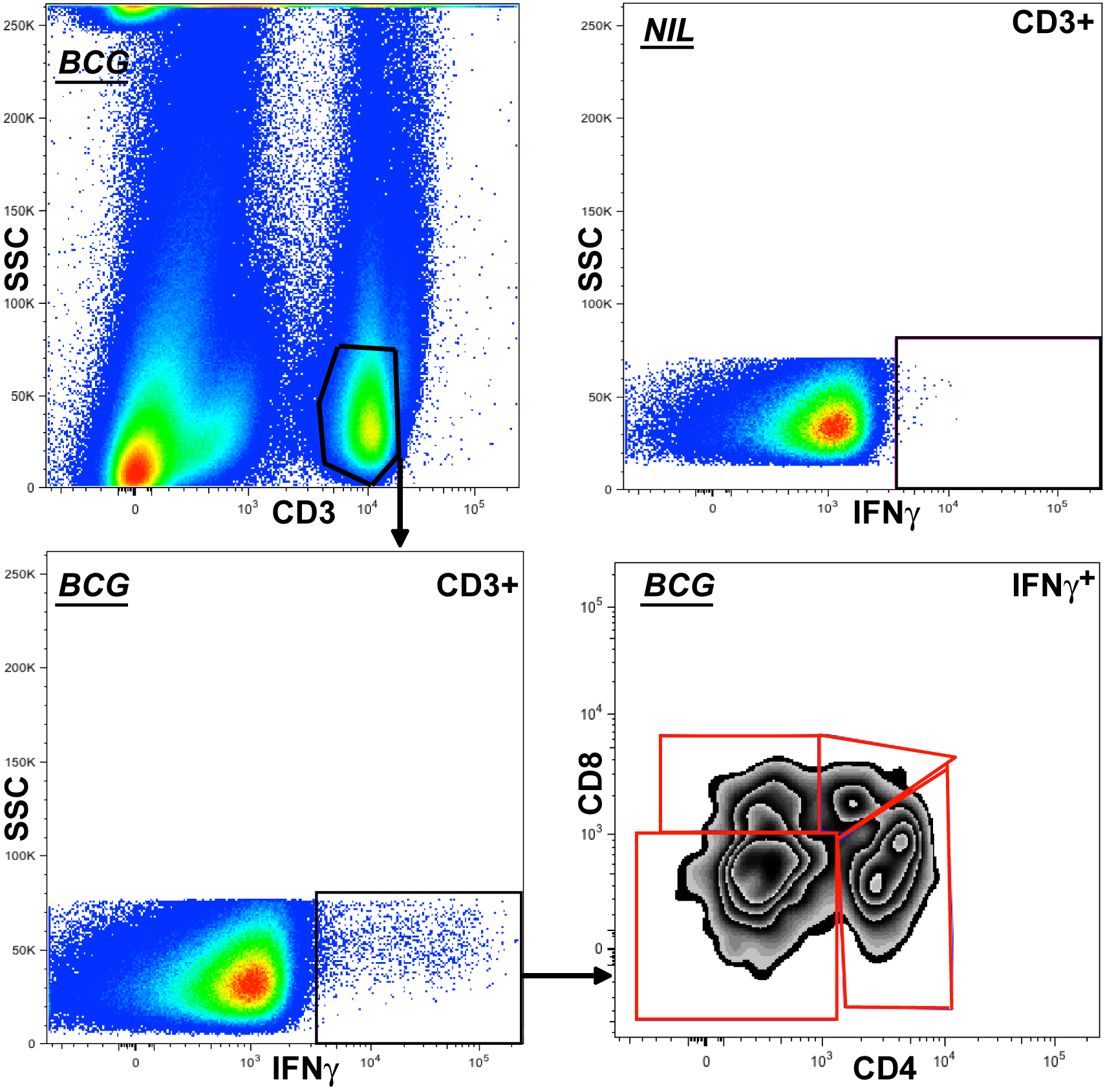

Supplement: Figure S1 — Gating strategy to select IFNγ-expressing cells within the CD3 T cell population. The IFNγ positive gate was set using Nil-stimulated samples (top right panel). In BCG-stimulated samples (bottom panels), CD8 and CD4 expression was then analyzed on CD3+ IFNγ+ cells. (TIF) [file pone.0077334.s001.tif]

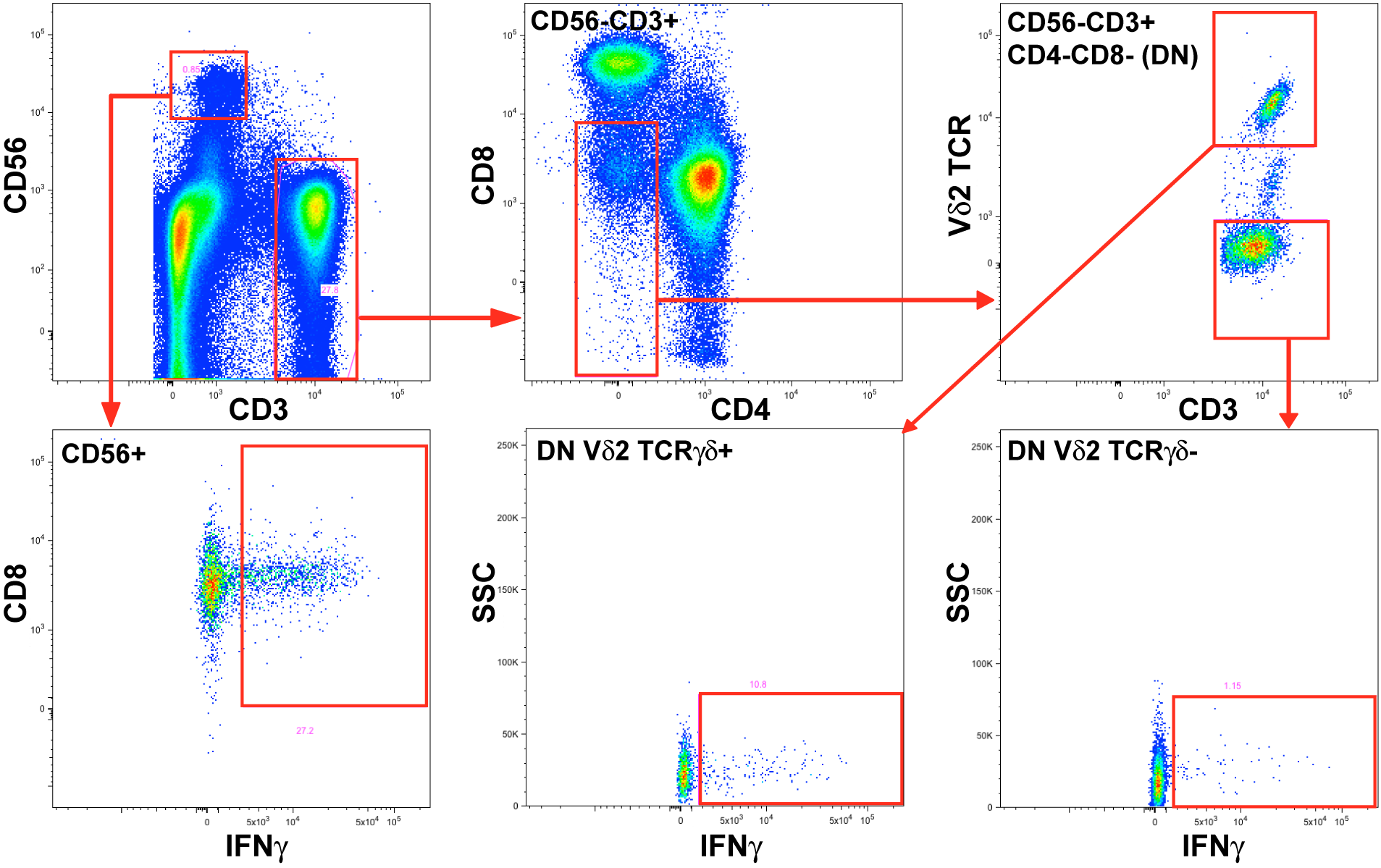

Supplement: Figure S2 — Gating strategy to select CD56+ NK cells and CD56-CD3+ T cells. Within the CD56-CD3+ cells, DN T cells were further gated into DN Vδ2 TCRγδ+ and DN Vδ2 TCRγδ- populations. Bottom panels show IFNγ expression in NK, DN Vδ2 TCRγδ+ and DN Vδ2 TCRγδ- cells. Note, for clarity, gating of CD4+, CD8+ and CD4+CD8+ T cells within CD56-CD3+ gate is not shown. (TIF) [file pone.0077334.s002.tif]
